# Behavioral Disorders in Mares with Ovarian Disorders, Outcome after Laparoscopic Ovariectomy: A Case Series

**DOI:** 10.3390/vetsci10080483

**Published:** 2023-07-25

**Authors:** Paola Straticò, Jasmine Hattab, Giulia Guerri, Augusto Carluccio, Lorenza Bandera, Gianluca Celani, Giuseppe Marruchella, Vincenzo Varasano, Lucio Petrizzi

**Affiliations:** Department of Veterinary Medicine, University of Teramo, 64100 Teramo, Italy; pstratico@unite.it (P.S.); jhattab@unite.it (J.H.); acarluccio@unite.it (A.C.); lbandera@unite.it (L.B.); gcelani@unite.it (G.C.); gmarruchella@unite.it (G.M.); vvarasano@unite.it (V.V.); lpetrizzi@unite.it (L.P.)

**Keywords:** behavior, estrus, granulosa cell tumor, ovariectomy, equine

## Abstract

**Simple Summary:**

Horse misbehavior is a common complaint from owners. Among these, estrus-related causes are common. Finding the specific underlying cause is not easy, nor is treating it. Clinical records of horses referred for laparoscopic ovariectomy for unwanted behavior were retrospectively reviewed and their owners were interviewed to determine the degree of improvement after surgery. The most common behaviors leading to referral were increased sensitivity on both flanks and general riding problems. Following surgery, most owners reported a significant improvement in behavior, with a reduction in severity or complete disappearance. Almost half of the removed ovaries had histologic characteristics of granulosa cell tumors. In the case of unilateral removal, normal estrous cycles were restored, and pregnancy was achieved within one year of surgery.

**Abstract:**

Owner complaints of estrus-related behavior in mares are a common cause of referral for laparoscopic ovariectomy. Granulosa cell tumors are a common neoplastic condition affecting the equine ovary, causing behavioral changes at rest and reduced performance. The reported success rate of ovariectomy in treating behavioral disorders is 64–86%. The aim of this study was to retrospectively evaluate the long-term follow-up of laparoscopic ovariectomy in mares in our case series, focusing on the owner’s perspective of the behavior of the mares after surgery. In addition, the histopathological features of the removed ovaries were investigated. The clinical records of mares that underwent laparoscopic ovariectomy between 2015 and 2022 were retrospectively reviewed. Owners complaining of poor behavior were interviewed about the main behavioral problem leading to referral and its eventual resolution after surgery. Eleven mares were included. The most common complaints were increased sensitivity on both flanks (10/11, 91%) and general riding problems (9/11, 82%). In 5/11 cases (45%), both ovaries were removed by laparoscopic ovariectomy. Histopathologic findings consistent with GCT/GTCT were found in five out of six examined ovaries (five granulosa theca cell tumors, GTCT). According to owner interviews, the scores assigned to each behavior improved significantly after surgery, regardless of histological findings. Although many factors can influence the behavior of horses, granulosa cell tumors (GCTs) proved to be a common cause and, as reported by the owners, ovariectomy resulted in improvement or complete resolution of the abnormal behavior.

## 1. Introduction

Estrus is a well-documented cause of undesirable behavior in female equids when they are ridden [1,2,3,4]. Trainers and owners often claim estrus as a potential distracting factor for their mares, especially when they are asked to perform at a high level. According to a survey conducted by the AAEP (American Association of Equine Practitioners) in 1996, 90% of respondents reported that estrus had a major impact on the athletic performance of their mares [1], causing performance level lower than expected. In the same survey, only 1% of respondents believed that their mare’s performance was not affected by ovarian activity. The estrus cycle seems to also affect mollies [4,5]. In a study, Heaton and colleagues reported that about 70% of investigated mollies showed estrus-related behavior, which was described as frequent urination, mouthing, excessive tail whipping, working performance issues, unwillingness to work, and tail whipping. In the case of mares, owners’ complaints relate to reluctance to engage the hind limb, poor behavior under the saddle, tail wagging, squealing, and clitoral eversion. In some cases, aggressiveness towards other horses or people is reported [1,5,6].

Although owners often complain about the behavior of their mares, a systematic approach to a definitive diagnosis is often lacking and a true cause–effect relationship is not achieved. Questions about the time of the first occurrence of the complained behavior, its recurrence and intensity, cyclicity, and frequency of presentation should be asked. Its response to medications must be also investigated. Back pain related to increased ovarian sensitivity is a well-documented aspect in mares. Moreover, some authors suggest that steroid hormones level and their fluctuations during the cycle may also affect muscle function [7,8]. In fact, increased general motor activity and hyperexcitability are also described in mares, as in other species such as rats and cows, as an effect of estrogen secretion [9,10]. In addition to physiological steroid hormone activity, several pathological conditions of the ovary can affect the behavior of mares. These include non-neoplastic (abscesses, hematomas) [11,12] and neoplastic conditions (granulosa cell and granulosa theca cell tumors—GCT—teratomas or cystadenomas) [13]. In non-neoplastic conditions, abdominal discomfort due to ovarian enlargement and inflammation is the cause of the abnormal behavior, whereas in neoplasia there is an additional hormonal issue.

Granulosa cell tumors and GTCTs are common ovarian neoplasms accounting for 2.5% of all neoplasms in horses [14]; they affect mares of all ages, may be unilateral or bilateral, and are rarely malignant. They are responsible for stallion-like behavior, poor breeding performances and infertility, aggressiveness, prolonged estrus, and poor behavior under the saddle [13,15].

Medical treatment is usually ineffective or only temporarily effective in suppressing hormone secretion and improving behavior, so ovariectomy is the treatment of choice. Medical treatment aims to suppress ovarian activity and estrogen production by the ovarian theca and granulosa cells and can be achieved by oral altrenogest supplementation [16,17,18,19], intramuscular progesterone injections [16], or parenteral progesterone and estrogen implants [1]. The induction of a false pregnancy has also been attempted [20]. In addition, a GnRH vaccine provides immunization against GnRH and secondary suppression of sex hormone secretion [21,22,23,24]. The use of non-steroidal anti-inflammatory drugs (NSAIDs) may help to differentiate behavioral problems due to painful conditions from disorders of possible hormonal origin. Causes of unwanted behavior and reduced performance that are different from the reproductive system must be excluded such as urinary tract disorders (vaginitis, pneumo-vaginitis, cystitis, and urolithiasis) and low-grade musculoskeletal pain. Since among the main owners’ complaints, poor rideability is mentioned, rider factors must also be ruled out.

The surgical treatment of bilateral standing ovariectomy was first described in 1993 [25]. In recent decades, laparoscopy has become the technique of choice for both ovariectomy and cryptorchidectomy in equids due to the limited complications and avoidance of general anesthesia [26,27,28,29]. Compared to the open ovariectomy, hemorrhage, infection, adhesion, herniation and evisceration associated with surgery are less common [30,31], while postoperative recovery and return to athletic function are faster. Cosmetic results are also unremarkable. Despite its advantages, bilateral ovariectomy may not be the option of choice when mares of high genetic value are considered.

The aim of this report was to investigate the most common behavioral problems in mares referred for laparoscopic ovariectomy. The long-term effects of gonadectomy on behavior was also retrospectively investigated through a telephone questionnaire that was addressed to owners/trainer and the referring veterinarians. We hypothesized that surgical removal would produce a behavioral improvement both in the case of normal ovaries and in those with GCT/GTCT.

## 2. Materials and Methods

Mares referred to the Veterinary Teaching Hospital of the University of Teramo with a history of estrus-related disorders and treated by laparoscopic ovariectomy were included in this study. The clinical records of each patient were retrospectively reviewed for behavioral history, breed, age, attitude, and ovarian histopathology when available [32]. To assess the outcome, owners, riders, or handlers together with their referring veterinarian, were retrospectively interviewed and a telephone questionnaire was administered. They were asked to list the type of misbehavior (general problems when ridden, increased sensitivity at both flanks, attempts to bite other horses, attempts to kick humans, attempts to kick other horses, aggressive behavior when approached from behind, attempts to buck when at work, poor quality contact with the bit, unwillingness to engage the hindquarters, and attempts to rear when ridden), the time from presentation to referral (more or less than 12 months), and to score the misbehavior before and after surgical treatment on a scale of 1 to 10, with 1 being the least disruptive and 10 being the most disruptive. Questions were also asked about medical treatment attempted prior to surgery (altrenogest, non-steroidal anti-inflammatory drugs) and its effectiveness. They were also asked if a vulvoplasty (Caslick’s procedure) was attempted before referral and its efficacy. The last question was about the overall satisfaction with the procedure and whether they would suggest it to others. The length of follow-up was also recorded.

Laparoscopic ovariectomy was performed unilaterally if there was evidence of ipsilateral macroscopic abnormality (one enlarged ovary on rectal palpation or unilateral abnormal ultrasound appearance). Bilateral ovariectomy was performed in cases of persistent abnormal behavior without evidence of specific macroscopic ovarian abnormalities [33,34]. After surgical preparation of the flank, bilaterally for bilateral procedures or ipsilaterally for unilateral ovariectomies, the abdominal wall was injected with 20 mL of 2% lidocaine via anatomical landmarks: the first access was located halfway along a horizontal line connecting the last rib to the ventral border of the tuber coxae above the border of the *m. obliquus esternus abdominis*. The other two portals were located 3 cm proximal and 5 cm ventral to the first [35]. A 1.5 cm vertical stab incision was made through the abdominal wall at the level of the first landmark; through this, a blunt trocar and cannula (11 mm diameter, 20 cm length) were inserted into the abdomen. A 30°, 10 mm diameter, 58 cm long laparoscope was then inserted through the cannula at the first central portal and the abdomen was insufflated with CO_2_ to achieve adequate pneumoperitoneum (12–15 mmHg). Afterwards, the laparoscope was shifted to the dorsal portal (made through another 1.5 cm vertical stab incision through the abdominal wall), while the Ligasure forceps were introduced into the abdomen through the central portal. An additional cannula was placed 5 cm distal to the first one through the other portals.

Once visualized, the ovaries were grasped with a pair of laparoscopic grasping forceps, which were used to hold them while the ovarian pedicle was injected with 10–20 mL of 2% lidocaine through a laparoscopic injection needle and during transection of the mesovarium. At the end of the procedure, once one or both ovaries were excised and homeostasis assessed, the excised ovaries were removed through the ventral incision, which was conveniently enlarged. In the case of bilateral ovariectomy, each ovary was removed from the ipsilateral side.

The distal portal of the abdominal wall was closed by suturing the fascia and muscle with polyfilament absorbable suture material (Polysorb, USP 1) in a simple continuous pattern. The skin was closed using a surgical skin staple device. The proximal portals were sutured in one layer, including the cutis, using disposable skin staples.

Representative tissue samples were promptly fixed in 10% neutral buffered formalin, embedded in paraffin, and routinely processed for microscopic examination (hematoxylin and eosin stain).

A descriptive statistic of pre- and post-surgical scores assigned to each behavior in all patients was provided. Due to the distribution and type of data, non-parametric statistical tests were used. A Wilcoxon signed-rank test was used to assess significant differences between pre- and post-surgical values. The subset of patients diagnosed with GCT/GTCTs at histopathology was analyzed separately. The level of significance was set at *p* < 0.05.

## 3. Results

Eleven mares were included in the study from 2015 to 2022 (six Standardbreds, two ponies, two thoroughbreds, and one Quarter Horse). The median age at presentation was 5 years (range 4–13 years). The median follow-up to owners’ interviews was 20 months (range 6–96 months).

The most frequent complaints were increased sensitivity at both flanks (10/11, 90.1%), general riding problems (9/11, 82%), and unwillingness to engage the hind quarters when ridden (8/11, 73%) (Table 1). In 6/11 of cases (54.5%), the complained misbehavior arose less than 12 months before referral.

The attempted medical treatments were oral administration of altrenogest (0.044 mg/kg every 24 h) from the referring vet and parenteral flunixin meglumine (1.1 mg/kg every 24 h) (4/11, 36%). Satisfaction with the first treatment was complete (absence of complained behavior during treatment) in 1/4 cases (25%), and mild (most of the complained behaviors disappeared during treatment) in 2/4 cases (50%). When flunixin meglumine was administered (1/11, 9.1%), no effect could be appreciated according to the owner and the referring vet. Even when effective, recurrence of misbehavior was the common complaint as soon as medical treatment was suspended.

Caslick’s vulvoplasty was never attempted.

Bilateral ovariectomy was performed in 5/11 cases (45%), a right ovariectomy in 5/11 cases (45%), and in 1/11 cases (9%), a left ovariectomy was accomplished. Six out of 11 mares had histopathology results available (55%). Nine ovaries were histologically examined, from six mares (three from bilateral ovariectomy and three from unilateral ovariectomy). Of these, five GCT/GTCTs were diagnosed, three from unilateral ovariectomy and two from bilateral ovariectomy. In the latter case, the neoplasia was identified only in one ovary.

Ovarian neoplasia was detected in five out of six mares that had their ovaries examined with histopathology.

The score assigned to each behavior changed significantly after laparoscopic ovariectomy (LO) (Table 2).

In 3/6 cases (50%) of unilateral ovariectomy, a normal estrus cycle was restored which led to a successful pregnancy within one year from surgery.

Overall, owner satisfaction was excellent, with 10/11 (91%) of respondents recommending the treatment to others.

In mares histologically diagnosed with GCT/GTCT (5/11 cases), the mean age at presentation was 7.2 years (range 4–13 years). The median time to follow up at an owner interview was 20 months (range 96–6 months). The time from onset of clinical signs to referral was less than 12 months in 3/5 cases (60%). In mares diagnosed with GCT/GTCT, the most common complaints were general problems when ridden, increased sensibility on both flanks, biting or kicking other horses (4/5, 80%), kicking humans, bucking at work, pulling away from contact when asked to engage the hindquarters and unwillingness to engage (3/5, 60%) (Table 3).

The mean score assigned to each behavior was significantly higher before than after ovariectomy (Table 4).

## 4. Discussion

In this retrospective case series, we focused on the behavioral problems reported by the owners and the referring vets, and the perceived severity before and after surgical removal of one or both ovaries. All patients were referred for laparoscopic ovariectomy and underwent unilateral or bilateral surgery. This decision relied upon unilateral evidence of a macroscopic abnormality (one enlarged ovary on rectal palpation or unilateral abnormal ultrasound appearance) or the persistence of abnormal behavior without evidence of specific macroscopic ovarian abnormalities. In both cases, surgery was effective in reducing or eliminating the complained behavior at follow-up interviews with good overall satisfaction.

Behavioral disorders in mares that are attributed to ovarian activity or pathology are often nonspecific. Usually, the diagnosis is presumptive or suspected after successful treatment. The condition is described not only in mares but also in donkeys and mules [4,5]. Despite the different use of these animals, general undesired behavior is often reported with a vague description of the condition (aggressiveness towards humans or other horses/mules, unwillingness to work, reduced performance, tail wagging, squealing), and no attempt to identify the primary disorder is often tried. As in previous studies [3,32], in this study, the evolution of the most common complaints after surgery was obtained from owners, trainers, and their referring veterinarians by telephone interviews. The need to involve the referring veterinarian in the evaluation of behaviors before and after surgery was related to the technical aspect of some questions. Moreover, a professional opinion is more likely to reduce the bias coming from owners’ personal implications with the case. Since mainly sport horses were included in the study, the most common problems we identified were increased sensitivity at both flanks when ridden or during daily grooming, general riding problems, and unwillingness to engage the hindquarters.

Several causes can lead to unwillingness to work, difficulty in engaging the hindquarters, and aggressiveness towards other horses or humans. Several authors investigated the clinical presentation of back pain in sport horses, highlighting behavioral disorders in common with ovarian pathologies. Back pain has been in fact recognized to affect a wide variety of horses with varying degrees of severity, clinical manifestations, and primary lesions, including lesions of the thoracolumbar muscles and spine and supraspinous desmitis as well as ovarian enlargement of neoplastic or non-neoplastic origin. Rider’s related reason, inadequate schooling or training as well as learned behavior, can also be considered non-medical causes of misbehavior [36,37,38,39,40].

In the case of GCT/GTCTs, non-histological diagnosis is challenging as clinical signs are not always consistent between studies [41]. Even if a honeycomb appearance of the affected ovary can be seen on ultrasound, with or without ovarian enlargement [42,43], ultrasound alone is not enough to make a definitive diagnosis. The best option to diagnose GCT/GTCTs before surgical removal is a hormonal assay to measure the blood concentration of anti-Müllerian hormone; this is secreted by the granulosa cells in the ovary and has recently gained popularity as a potential biomarker for GCT/GTCTs. A sensitivity of about 98% is reported [43,44,45], although normal blood concentrations have been described even in the case of GCT/GTCT [41]. The reason for these uneven results may be related to the different thresholds used in different studies and probably to different stages of development of the neoplasia when present. What is clear from the literature is that anti-Müllerian hormone is more specific than inhibin or testosterone or their association with the diagnosis of GCT/GTCTs [46]. This is of particular value in the case of mares infertility in the absence of macroscopic ovarian abnormalities. In fact, mares with GCT/GTGC are often infertile because of inhibin, testosterone, and anti-Müllerian hormone secretion by the affected ovary [46]. If a pregnancy is desired by the owner, a preliminary assessment of hormonal concentration is mandatory, before deciding on surgery.

Among the medical treatments available to suppress estrus in mares we decided to investigate the use of altrenogest because it is the most common although not the most effective [18,47,48,49,50]. As it acts on the ovary to suppress estrus, it is thought to be a good drug to treat ovarian dysfunction. Its inhibitory effect is transient and estrus is restored within 5 days from interruption [17], suggesting its use when future breeding is considered [51]. However, it is approved for daily use for 10 days, and the restoration of ovarian activity at interruption may be seen not appropriate because together with estrus, undesirable behavior recurs. Moreover, it is expensive, and it is potentially dangerous for women [52,53,54]. Moreover, increased uterine susceptibility to inflammation is described [55,56]. Medical treatment had been tried before referral in 4/11 cases. In particular, altrenogest was mentioned in 3 cases with a mild grade of satisfaction in 2/3 cases (most of the complained behaviors disappeared during treatment) and good in 1/3 cases (absence of complained behavior during treatment). Only one mare with GCT/GTCT received the treatment with complete resolution of clinical signs. Non-steroidal anti-inflammatory drugs were used only in ¼ cases with no behavioral changes, ruling out undetected musculoskeletal pain or back discomfort coming from painful ovarian activity (cysts, enlarged ovaries, and large anovulatory follicles).

The reason to investigate the attempt of a Caslick’s vulvoplasty before surgery relies on its indication for the treatment of vaginitis and pneumovaginitis [57]. Its use was never referred to in this study.

In this case series, good overall satisfaction was observed retrospectively, with a significant reduction or complete disappearance of the reported behavioral problems. Unilateral ovariectomy was performed when a unilateral ovarian disorder was clinically diagnosed (enlargement and abnormal ultrasound appearance of the ovary). Granulosa cell tumor or granulosa theca cell tumor was clinically suspected before surgery in three horses and confirmed after histopathology. In the other two cases, GCT/GTCT was diagnosed by histopathology in one ovary after bilateral ovariectomy without preoperative clinical evidence of unilateral ovarian change. In these patients, bilateral resection was requested by the referring veterinarian after a presumptive diagnosis based on the response to the medical treatment that was attempted.

Behavioral improvement after surgery was significant after mono- or bilateral ovariectomy. In previous studies, laparoscopic ovariectomy showed resolution of the primary complaint in 95% of cases, with good overall satisfaction with the procedure [53]. However, the authors stated that owners had to be advised that not all estrus-related behavior may disappear. About this topic, they also described the persistence of lower intensity estrus manifestation in 30% of treated mares [53], similar to previous reports [25]. This is probably a result of the extragonadal secretion of steroid hormones [53,58]. In our experience, general problems when ridden and increased sensitivity at both flanks were still appreciated after surgery but with a significant reduction compared to presurgical scores.

In our study, the normal estrous cycle was restored in all cases of unilateral ovariectomy, with the achievement of a successful pregnancy in 3/6 cases. Although pregnancy is described in the presence of GCT [59,60,61,62], usually one of the complaints that are referred to is mare infertility, probably because of high inhibin concentration secreted by the affected ovary [41]. False negative results at hormonal assay are not infrequent because of the unpredictable endocrine activity of GCT/GCTC. So, the authors suggest not to rule out ovarian disorders in case of negative results. In the case series that was here described, even though some pregnancies were achieved, it is not known whether, in those mares that did not have a pregnancy, this was not desired by the owners or attempted and not obtained because of persistent hormonal dysfunction.

The time from onset of abnormal behavior to referral was less than 12 months in 54.5% and 60% of cases in the total population and in GCT-positive patients, respectively. It could be so hypothesized that the time of presentation and severity of clinical signs may not differ among neoplastic and non-neoplastic ovaries. However, the lack of histopathology in 5/11 cases makes not possible to draw any conclusion about this aspect, because of a possible underestimation of the incidence of GCT in the population.

Our results should improve knowledge of the management of undesirable behavior in mares. However, some limitations must be taken into account when interpreting them, such as the sample size, the lack of histopathology in almost half of the mares, as well as an endocrine panel and ultrasound data prior to surgery. Moreover, the retrospective nature of the study itself may be considered a limitation.

Telephone interviews or questionnaires are commonly used to retrospectively investigate owners’ opinions on specific conditions [3,32,53]. A placebo effect and a different personal perception of the severity of the disorders are not uncommon [3], especially in cases of not experienced owners or trainers. Assessing performance before and after treatment by the same person allows for a more objective assessment of improvement. To reduce the bias coming from this aspect we involved the opinion of the referring veterinarian. Practitioners are not emotionally involved in the clinical case and are able to give more detailed responses also to technical questions that may not be exhaustive when given by the owners.

## 5. Conclusions

In this retrospective case series, we described the most common behavioral disorders in mares and the effectiveness of uni/bilateral laparoscopic ovariectomy in treating them. The most frequent complaints that we described were increased sensitivity at both flanks, general riding problems, and unwillingness to engage the hind quarters when ridden. Infertility was also described. Granulosa cell tumor or granulosa teca cell tumor was identified in most of the ovaries that were histologically examined. In these cases, common behaviors were also biting or kicking other horses, kicking humans, bucking at work, pulling away from contact when asked to engage the hindquarters and unwillingness to engage. Uni or bilateral laparoscopic ovariectomy was effective in eliminating or reducing the severity of the symptoms both in neoplastic and non-neoplastic ovaries. After unilateral ovariectomy, pregnancy was achieved. Overall satisfaction was good in all cases. As owners report an improvement in abnormal behavior in mares after ovariectomy, and abnormal behavior is rarely associated with increased AMH, inhibin and inhibin B [63], we can hypothesize that in some mares normal levels of sexual hormones can induce misbehavior. Although there is growing evidence of the beneficial effects of ovariectomy for the treatment of moody mares, care must be taken in case of selection and in the establishment of a cause–effect relationship.

## Figures and Tables

**Table 1:** Frequency of behavioral expression as referred by owner/trainers in the whole sample. Values are expressed as ratio and percentage.

Behavior	Frequency of Expression
General problems when ridden	9/11 (82%)
Increased sensitivity at both flanks	10/11 (91%)
Tries to bite other horses	6/11 (55%)
Kicks humans	6/11 (55%)
Kicks other horses	8/11 (73%)
Aggressive behavior when approached from behind	5/11 (45%)
Bucks when at work	7/11 (64%)
Poor quality contact with the bit	7/11 (64%)
Unwilling to engage the hindquarters	8/11 (73%)
Rears when ridden	4/11 (36%)

**Table 2:** Preoperative and postoperative score assigned by owners/trainers to the different behaviors in the whole sample (values are expressed as median and range; Wilcoxon signed-rank test; statistical significance for *p* < 0.01).

Behavior	Preoperative Score	Postoperative Score	*p*-Value
General problems when ridden	9 (6–10)	1 (0–8)	0.00003
Increased sensitivity at both flanks	8 (7–10)	1 (0–6)	0.000001
Tries to bite other horses	9.5 (8–10)	0 (0–8)	0.0007
Kicks humans	8 (6–9)	0 (0–8)	0.005
Kicks other horses	10 (4–10)	0 (0–8)	0.00002
Aggressive behavior when approached from behind	8 (7–10)	1 (0–8)	0.004
Bucks when at work	8 (8–10)	0 (0–8)	0.00005
Poor quality contact with the bit	7.5 (7–10)	0 (0–6)	0.00001
Unwilling to engage the hindquarters	9 (7–10)	0.5 (0–6)	0.0003
Rears when ridden	9 (7–10)	0 (0–7)	0.0003

**Table 3:** Frequency of behavioral expression as referred by owner/trainers in the subset of patients with GCT/GTCT. Values are expressed as ratio and percentage.

Behavior	Frequency of Expression
General problems when ridden	4/5 (80%)
Increased sensitivity at both flanks	4/5 (80%)
Tries to bite other horses	4/5 (80%)
Kicks humans	3/5 (60%)
Kicks other horses	4/5 (80%)
Aggressive behavior when approached from behind	2/5 (40%)
Bucks when at work	3/5 (60%)
Poor quality contact with the bit	3/5 (60%)
Unwilling to engage the hindquarters	3/5 (60%)
Rears when ridden	2/5 (40%)

**Table 4:** Preoperative and postoperative score assigned by owners/trainers to the different behaviors in the subset of patients with GCT/GTCT (values are expressed as median and range; Wilcoxon signed-rank test; statistical significance for *p* < 0.01).

Behavior	Preoperative Score	Postoperative Score	*p*-Value
General problems when ridden	8.5 (6–9)	1 (0–4)	0.001
Increased sensitivity at both flanks	8 (7–10)	1.5 (0–2)	0.0001
Tries to bite other horses	9 (8–10)	0 (0–1)	0.00001
Kicks humans	8 (8–9)	1 (0–1)	0.00008
Kicks other horses	9 (8–10)	0 (0–1)	0.0001
Aggressive behavior when approached from behind	8 (7–9)	0.5 (0–1)	0.04
Bucks when at work	8 (8–10)	0 (0–0)	0.005
Poor quality contact with the bit	8 (7–8)	0 (0–1)	0.00009
Unwilling to engage the hindquarters	9 (7–10)	0 (0–1)	0.005
Rears when ridden	7 (7–10)	0 (0–1)	0.01

## Data Availability

The data presented in this study are available on request from the corresponding author. The data are not publicly available due to ethical and privacy reasons.
